# Drivers of green purchasing behaviour: a systematic review and a research agenda

**DOI:** 10.12688/f1000research.140765.1

**Published:** 2023-10-09

**Authors:** Nuryusnita Yusoff, Mazni Alias, Norhazlin Ismail

**Affiliations:** 1 Faculty of Management, Multimedia University, Cyberjaya, Selangor, 63000, Malaysia

**Keywords:** green purchasing behaviour, green procurement behaviour, sustainable procurement behaviour, sustainable procurement, theme analysis

## Abstract

**Background:** Green purchasing is an important aspect of sustainable consumption, which decreases society’s environmental effect. Although numerous research has been conducted to investigate the determinants of green buying behaviour, there has been a lack of effort in comprehensively analysing these findings. The purpose of this study is to examine the available literature on the factors that influence green purchasing behaviour.

**Methods:** The review focused on empirical research published in peer-reviewed English-language publications between 2017 and 2021 in Web of Science and Scopus. The research took place from May to June 2021. Mixed Methods Appraisal Tool (MMAT) is used to assess the risk of bias in systematic literature reviews.

**Results:** 41 articles were included, with significant focus on the retailing sector. Most of these studies were centred in Asian countries, primarily China and India. The Theory of Planned Behaviour was the most prominent, appearing 15 times, followed by the Theory of Reasoned Action (seven times). Analysis identified five main themes and 15 sub-themes related to green purchase behaviour drivers. These themes were categorized by occurrence: People (34 papers), marketing (13), knowledge (12), environment (12), and influence (nine). The dominant driver was people (34 studies), encompassing sub-themes including motivation (three), perception (eight), behavioural (13), and psychographic characteristics (10).

**Conclusions:** This study has given an overview of the present status of green purchasing behaviour, which serves as a foundation for future studies and guidance for policymakers and practitioners. However, it does not include unpublished materials and non-English papers. Secondly, it focuses on articles from two databases within the last five years which doesn’t encompass all article types, prompting the need for future exploration. Thirdly, extending the review’s time frame could unveil more pronounced GPB patterns. Lastly, although all eligible papers were assessed based on criteria, the chance of overlooking some papers is acknowledged.

## Introduction

Unsustainable levels of consumption around the world are causing serious environmental problems, prompting community to rethink their traditional purchasing behaviour to achieve environmental sustainability ( Jaiswal and Kant, 2018; Todorovic, 2018). For example, the usage of single used plastic (microplastics) that cause marine plastic pollution and has become a significant environmental concern for governments ( Xanthos and Walker, 2017). An increasing issue about microplastics is that they can penetrate the human food chain by consumption of fish, shellfish, and filter feeders ( Chang, 2015), potentially adverse effects on human health ( UNEP, 2015; GESAMP, 2016). Therefore, consumers must adjust their purchasing habits to address the harmful environmental and human health implications of pollution. Changing one’s consumption habits can help to alleviate environmental problems and encourage sustainable production ( Xanthos and Walker, 2017). For example, by purchasing ecologically friendly packaging, shoppers can put pressure on retailers to migrate from traditional packaging methods to eco-friendly packaging methods by employing less damaging packaging materials. This, in turn, minimises worldwide marine plastic pollution.

As a result of growing concern about environmental damage, people have started to convert their environmental awareness into pledges to buy ‘green’ items ( Haque, Yamoah and Sroka, 2020; Sheng *et al.,* 2019) which can be called green purchasing behaviour (GPB). Green consumer behaviour, in other terms, refers to the behaviours of a person who considers social impacts when making purchases ( Sharma and Joshi, 2017). GPB is critical in minimizing the environmental impact of consumed goods to meet the importance of green consumption. Many researchers (e.g. Ahn, Kim and Kim, 2020; Elsantil and Hamza, 2019 and Anantharaman, 2018) have aimed to figure out why people buy green products by looking at the assembly process, methods, components used, the ecological consequences, and how businesses are involved ( He *et al.,* 2021).

Modifying one’s purchasing behaviour can aid in the reduction of environmental issues and the promotion of sustainable production ( Ahn, Kim and Kim, 2020). Subtle changes in daily behaviour can pave the road for a more sustainable future. As identified by Elsantil and Hamza (2019) and Anantharaman (2018), recently, there has been a big rise in the number of people in study on individual behaviour modification and sustainable consumption. Finding a link between environmentally conscious customer attitudes and GPB from the larger green marketing field, is a new area of research ( Trivedi, Patel and Acharya, 2018).

Even though various research has been conducted to investigate the causes of GPB, there has been an absence of initiative in comprehensively assessing the findings. In the present year, no systematic reviews of green purchasing behaviour have been conducted. There is literature review on GPB by Nadeem, Mohamad and Abdullah (2017), provided an overview of effects of personal values, leadership style and awareness on GPB meanwhile ( Witek and Kuźniar, 2021) investigating how socio demographic factors explain consumers’ GPB. Even though the latter briefly reviewed published studies on GPB drivers, the present article will systematically review drivers of GPB, using PRISMA 2020 guidelines.

Therefore, the purpose of this study is to (i) to give a complete and systematic analysis of the drivers that significantly influence consumers’ GPB; and (ii) highlight the research trend and diverse issues by conducting a thematic analysis of the chosen articles. This objective was met based on qualitative data analysis, we conducted a systematic literature review (SLR), descriptive and thematic data analysis. Preferred Reporting Items for Systematic Reviews and Meta-Analyses 2020 and Mixed Methods Appraisal Tool (MMAT) Version 2018 ( Nha *et al.,* 2018) were used as framework to carry out the SLR.

The research questions are as follows. First, how can we systematize and identify advances in analytical areas of GPB research by going through key papers, theories, methodologies, and variables of interest in existent literature. Second, how can we develop a thematic theme for analyzing GPB that examines the drivers. Third, what are the directions for future research and its implications.

### Literature review

The progressive destruction of the natural environment because of human exploitation necessitated the introduction of the idea of sustainable development. As a result, protecting the natural environment and resources for future generations has become a global necessity, and one method is to engage in green purchasing ( Ali *et al.,* 2020; Giao, 2020). Since the 1990s, green purchasing has been perceived as an effective tool in lessening environmental burdens pertinent to the human activities of product production and consumption ( Ho, Dickson and Chan, 2010; Malatinec, 2019). The strategic role of purchasing and supply as a lever for sustainable development is reflected in an increase in the prevalence of research on responsible purchasing and supply ( Ridzuan Kushairi, 2019), a greater policy emphasis on green and sustainable procurement in the public sector ( Igarashi, de Boer, and Pfuh, 2017), and private-sector efforts to improve the environmental and social performance of firms and their supply chains ( Mashele and Chuchu, 2018).

Previous research have concentrated mostly on the core values, opinions, and intentions of customers while purchasing eco-friendly goods in order to describe sustainable consumption ( Liobikienė and Bernatonienė, 2017). Various research have been undertaken to examine the primary elements influencing consumers’ long-term purchasing behaviour, including perceived behavioural control ( Müller *et al.,* 2021; Zahan *et al.,* 2020), attitude ( Trivedi *et al.,* 2018; Zahan *et al.,* 2020), environmental concern ( Wang, Wong, and Narayanan Alagas, 2020), environmental responsibility ( Joshi and Rahman, 2019), marketing instruments ( Sari *et al.,* 2020), environmental knowledge ( Wang *et al.,* 2020), subjective norms ( Joshi and Rahman, 2017) and social norms ( He *et al.,* 2021). Furthermore, earlier studies on green purchasing have investigated many personal, psychological, and societal elements that influence customers’ environmentally and socially conscious purchasing ( Liobikienė and Bernatonienė, 2017). These conceptions are difficult to interpret, yet they are crucial in behavioural and cultural psychology. Many scholars have explored and analysed these constructs to analyse consumer green purchasing decisions. Regardless their environmental awareness and worries, consumers’ attitudes and behaviours differ when it comes to purchasing products. Most researchers have confirmed that green consumer behaviour is a future behaviour that contributes to environmental protection ( Giao, 2020; Naz *et al.,* 2020).

Implementation of green purchasing practice is subjected to typical reasons, and the effectiveness of this practice is determined by key factors. It is therefore the aim of this study is to examine green purchasing behaviour to explain how various factors influence green purchasing behaviour. This study provides an important foundation to understand green purchasing behaviours and, in turn, analyse critical elements that influence them. Understanding these critical aspects can assist relevant government agencies in developing effective policies to encourage developers to use green purchasing, so promoting sustainable development.

## Methods

The mechanism for retrieving articles relating to GPB drivers is covered in this section. Preferred Reporting Items for Systematic Reviews and Meta-Analyses 2020 and Mixed Methods Appraisal Tool (MMAT) Version 2018 ( Nha *et al.,* 2018) were the strategies employed to carry out the SLR.

### Preferred Reporting Items for Systematic Reviews and Meta-Analyses

The Preferred Reporting Items for Systematic Reviews and Meta-Analyses (PRISMA) 2020 Statement, which superseded the 2009 statement and incorporates new reporting criteria that reflect improvements in techniques for finding, selecting, assessing, and summarizing research, guided this assessment ( Page *et al.,* 2021). PRISMA 2020 is intended for use in systematic reviews with or without synthesis (for example, pairwise meta-analysis or other statistical synthesis approaches). This methodology has been used by several studies in literature medical research such as Bekele, Mechessa and Sefera (2021), Puljak and Pieper (2022) and in social sciences with Vejaratnam, Mohamad and Chenayah (2020).

### Systematic review process

The journal databases utilised in this SLR were Web of Science and Scopus. These databases were selected due to the large compendium of documents available from Emerald, Elsevier, Springer, and Taylor and Francis. Each research string has the following keywords: “green procurement behavi*r” or “sustainable procurement behavi*r” or “sustainable purchasing behavi*r” or “green purchasing behavi*r”, or “pro-environmental purchasing behavi*r.” Keywords relevant to this study such as “Purchas*” (buying and purchase) were also included since it has a very similar meaning to procurement and is used by various authors. Within this research, ‘environmental’ has been included because it is synonymous with ‘green’.

We will now go over the screening approach in detail. First, we chose the keywords for our study (the “*” symbol was used at the end of some phrases to broaden the range of viable studies, as many articles use slightly different terms for the same idea, such as “sustainable” instead of “sustainability”). We chose one word linked to sustainability/corporate social responsibility (i.e., “csr” keywords) to broaden the potential list of studies that might be related to our study questions: “sustainab *”, “environment *”, “green”, and “corporate social responsibility”.

The qualifying conditions for this review were empirical research published in peer-reviewed full-length publications written in English between 2017 and 2021. The chosen publishing time frame was determined by the need to keep the sources trending. The literary research session lasted from May to June of 2021. We did not examine review articles, book series, books, book chapters, or conference proceedings since we only intended to examine primary sources containing empirical data. A total of 415 articles were found after searching both databases. After the exclusion, there were 359 articles left. After removing 77 duplicates from both databases, the qualifying process was reduced to 282 articles.

**Figure 1.**
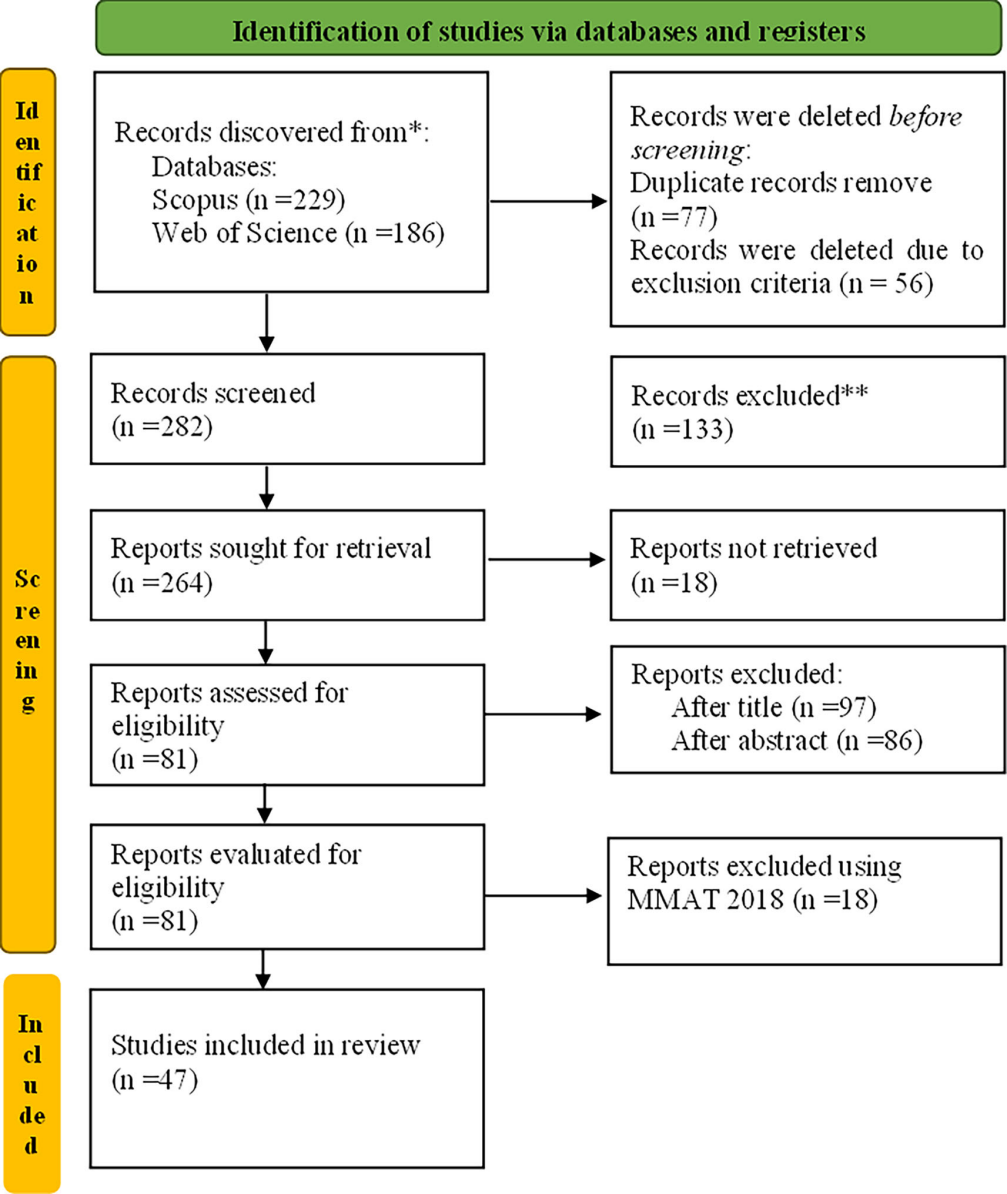
The study's flow diagram PRISMA 2023.

Abstracts were checked for relevance at this point, and some papers were read entirely to identify research that empirically studied factors of GPB. Articles about green purchasing were only considered if they investigated human behaviour. Some articles, such as Morales-Contreras *et al.* (2019) looked at barriers to GPB; nevertheless, hurdles to GPB discovered in the research were also documented. Eighteen articles were omitted because they were not retrievable. After screening the related articles based on the title and abstract, 81 papers proceed to the next screening using Mixed Methods Appraisal Tool (MMAT) Version 2018 by ( Nha *et al.,* 2018). MMAT was chosen because the papers listed contain qualitative, quantitative, and mixed methods. This method resulted in the identification of 41 publications for qualitative examination. As a result, 41 remaining articles were considered for the review. Similar studies have suggested a comparable number of articles for systematisation ( Benevene and Buonomo, 2020; Gimenez and Tachizawa, 2012; Jabbour, 2013). Figure 1 shows the stages of articles selection that adapted based on Page *et al.,* 2021. The classification and coding of these articles were performed as described in the next section.

### Data abstraction and analysis

**Figure 2.**
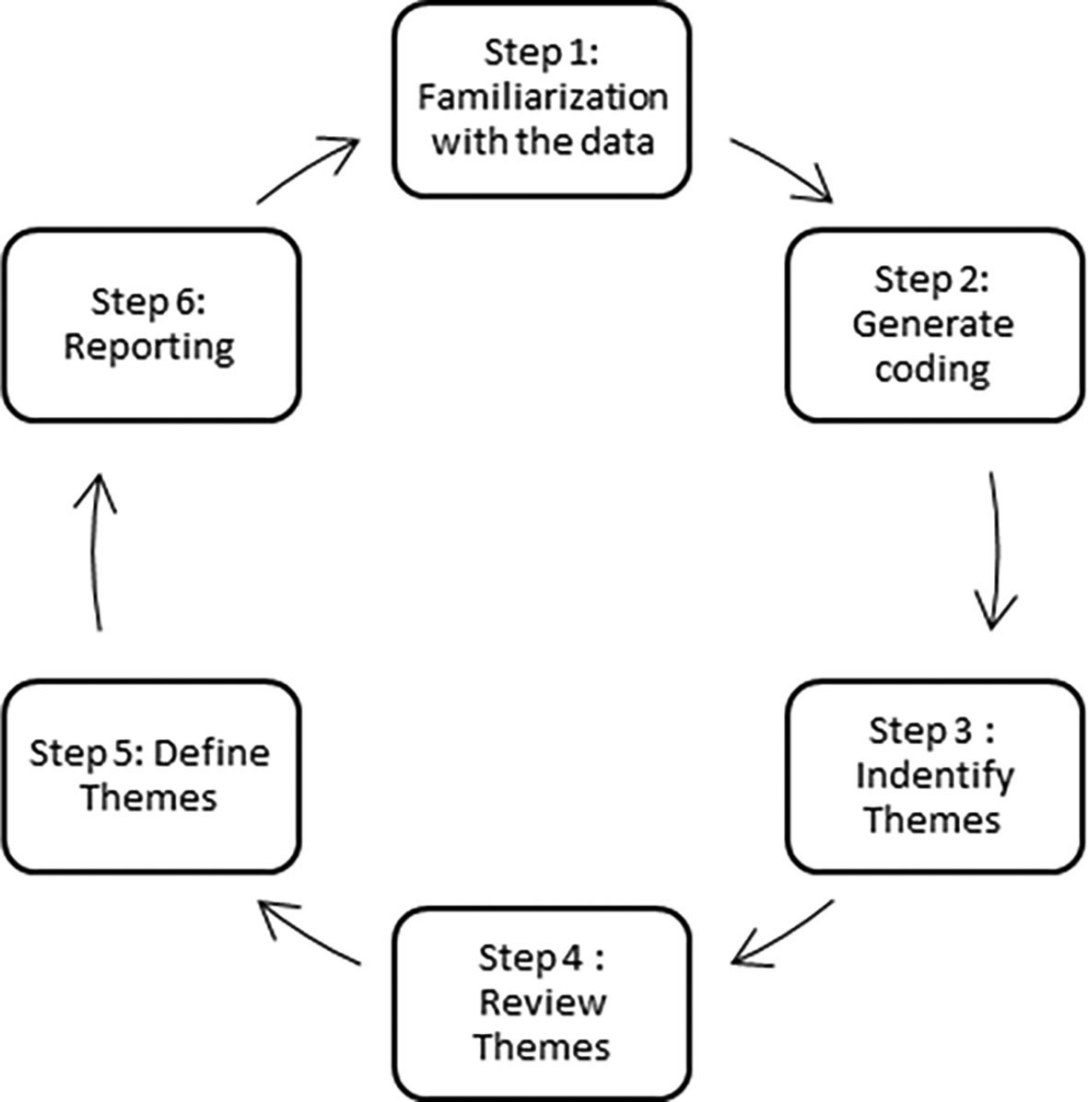
Six-Step framework: thematic analysis by Braun and Clarke (2006).

**Figure 3.**
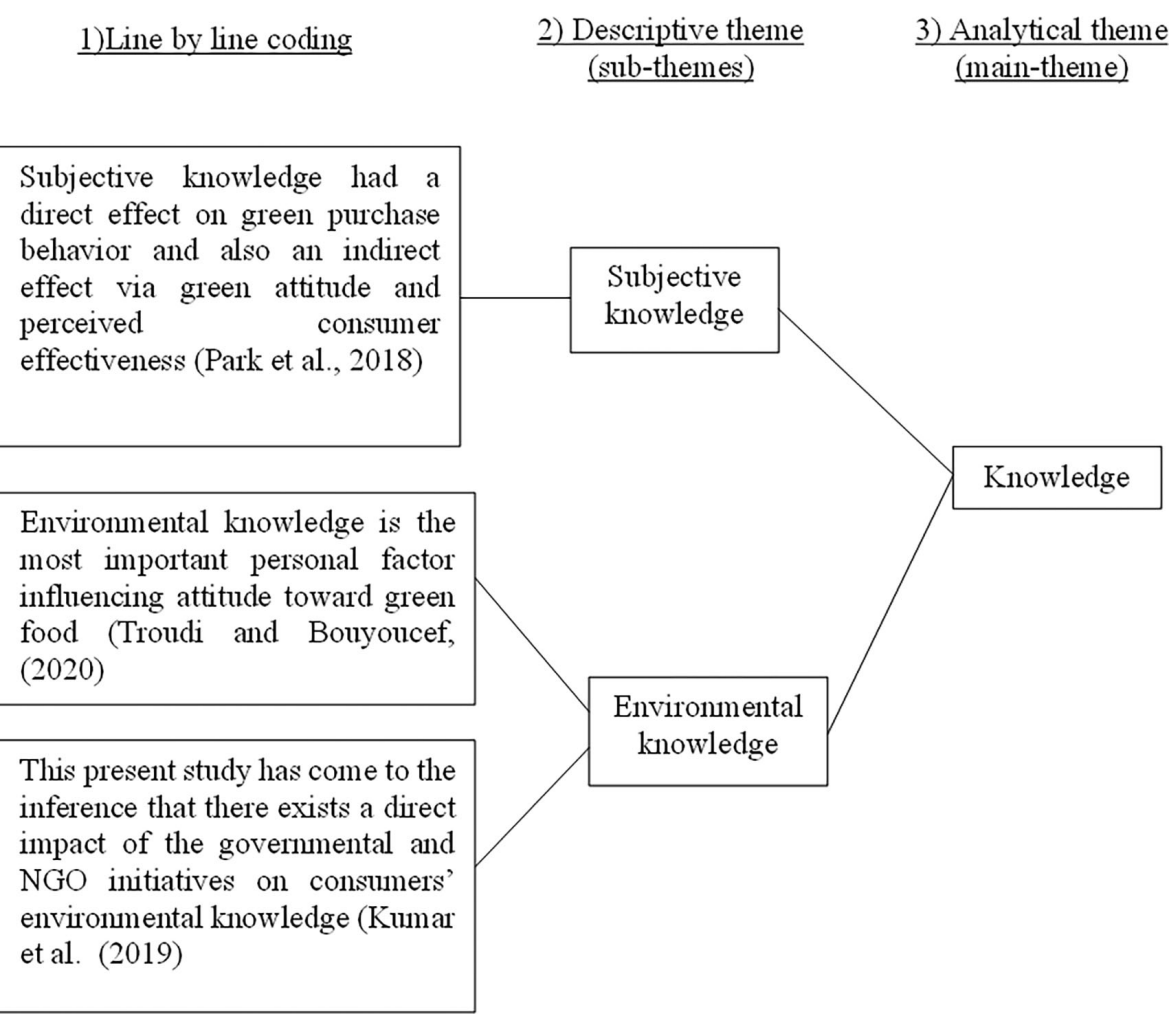
The coding process.

The qualitative approach begins with an abductive process known as template or thematic analysis, which is used to build richer theoretical views than those already available in literature. Thematic analysis was chosen because it is an extended assessment with the objective of going beyond a data summary, attempting to build on key themes and order and organise the literatures under these topics ( Xiao and Watson, 2019). Thematic evaluations with a framework have been shown to be more acceptable since they have a more stable structure ( Paul and Criado, 2020). Therefore, Braun and Clarke (2006) six step framework are adopt and adapt to form a systematic thematic analysis, illustrated in Figure 2 below:

After we obtained 415 articles from the database queries, we read through the entire articles set before we begin the coding, as we get ideas, identification of possible patterns will be shaped as we read through. The idea is to be familiar with all aspects of the data (Step 1). The coding procedure was broken down into three phases. First, each of the selected articles was coded line by line to obtain statements on GPB drivers. Following that, sentences that highlight comparable points were grouped together to form a descriptive theme, or subtheme (Step 2 until Step 4) as shown in Figure 3.

The final stage was grouping sub-themes into analytical themes, also known as major themes (Step 5 and 6). Five relevant sets of drives (themes) and 15 sub-themes emerged from the coding process. The expert assessment was done by a human behaviour expert and an environmental management expert to guarantee the validity of the themes and sub-themes. These codes are then collated into the main and initial sub-themes of the hierarchy in as Table 1 below:

**Table 1.** List of major and sub-theme.

Major theme:	People			
Sub-theme:	motivation	perception	behavioural	psychographic characteristic
Major theme:	Environment			
Sub-theme:	environmental concern	environmental responsibility	environmental practices	environmental education
Major theme:	Marketing			
Sub-theme:	market instrument	market condition	Supplier	
Major theme:	Influence			
Sub-theme:	subjective norms	social norms		
Major theme:	Knowledge			
Sub-theme:	subjective knowledge	environment knowledge		

## Results

The results were broken down into two sections: 1) descriptive analysis and 2) thematic analysis. The descriptive analysis results give bibliographic data and assist in contextualising the outcomes of the thematic identification and material evaluation processes. Thematic analysis is the process of grouping studies into research guiding topics based on similarities and trends discovered.

### Descriptive analysis

Notably, most of the studies are retailing (22 studies). Several studies concentrated on business sectors i.e., education (two studies), food system (two studies), hotel (three studies), personal care product (two studies), construction (two studies), and housing (two studies). Besides that, one study are from the government, automotive, recycling company, and battery industries as shown in Table 2. In industrialized countries, 15 studies were undertaken, however in poor countries, the number of studies was nearly doubled, with 26 studies.

**Table 2.** List of sectors involved in this study.

Sector	Author	Number of studies
Education	Ahn *et al.* (2020)	2
	Uddin and Khan (2018)	
Food system	Laureti and Benedetti (2018)	2
	Troudi and Bouyoucef (2020)	
Hotel	Morales-Contreras *et al.* (2019)	3
	Nimri *et al.* (2020)	
	Wang *et al.* (2020)	
Personal care product	Al Mamun *et al.* (2020)	2
	Eberhart and Naderer (2017)	
Retailing	Ahamat *et al.* (2018)	22
	Al-Adamat *et al.* (2020)	
	Amoako *et al.* (2020)	
	Caniëls *et al.* (2021)	
	Choi and Johnson (2019)	
	Giao (2020)	
	Jaiswal and Kant (2018)	
	Joshi and Rahman (2019)	
	Kumar *et al.* (2017)	
	Liao *et al.* (2020)	
	Mohd Suki and Mohd Suki (2019)	
	Müller *et al.* (2021)	
	Park and Sohn (2018)	
	Sari *et al.* (2020)	
	Sharma *et al.* (2020)	
	Sheng *et al.* (2019)	
	Siyavooshi *et al.* (2019)	
	Trivedi *et al.* (2018)	
	Van Tonder *et al.* (2020)	
	Varela-Candamio *et al.* (2018)	
	Wang *et al.* (2019)	
Construction	Yang *et al.* (2020)	2
	Yang *et al.* (2019)	
Electronic	Ahmad and Zhang (2020)	2
	Ali *et al.* (2019)	
Housing	Zahan *et al.* (2020)	2
	Zhang *et al.* (2019)	
Automotive	He *et al.* (2021)	1
Battery industry	Zhang *et al.* (2018)	1
Government	Leal *et al.* (2020)	1
Recycling company	Onel (2017)	1

In terms of developing countries, the survey found that Asian nations were the most well-represented (24 studies). China was the country with the most studies (10), followed by India (eight studies) as shown in Table 3. The fact that most of the studies were undertaken in developing nations contradicts a prior conclusion by Ahamat, Ahmad and Mohd (2018) who determined that very little research on consumer demand for environmentally friendly commodities in developing countries had been conducted. As pointed out by Wang *et al.* (2020) and Mohd Suki and Mohd Suki (2019), GPB research are quite common in underdeveloped countries. One of the motivations on this motion probably because Asian consumers are considerably less conscious of environmental sustainability than consumer in developed countries ( Ahamat *et al.,* 2018).

**Table 3.** List of articles sort according to countries.

Country	Authors		Total
Algeria	Troudi and Bouyoucef (2020)		1
Australia	Nimri *et al.* (2020)		1
Bangladesh	Zahan *et al.* (2020)		1
Cambodia	Liao *et al.* (2020)		1
China	Ahmad and Zhang (2020)	Wang *et al.* (2019)	10
	Ali *et al.* (2019)	Yang *et al.* (2019)	
	He *et al.* (2021)	Yang *et al.* (2020)	
	Sheng *et al.* (2019)	Zhang *et al.* (2018)	
	Wang *et al.* (2020)	Zhang *et al.* (2019)	
Germany	Eberhart and Naderer (2017)		1
Ghana	Amoako *et al.* (2020)		1
India	Jaiswal and Kant (2018)	Troudi and Bouyoucef (2020)	8
	Joshi and Rahman (2019)	Joshi and Rahman (2017)	
	Kumar *et al.* (2017)	Sharma *et al.* (2020)	
	Trivedi *et al.* (2018)	Uddin and Khan (2018)	
Indonesia	Sari *et al.* (2020)		1
Iran	Siyavooshi *et al.* (2019)		1
Italy	Laureti and Benedetti (2018)		1
Spain	Morales-Contreras *et al.* (2019)	Varela-Candamio *et al.* (2018)	2
Jordan	Al-Adamat *et al.* (2020)		1
Malaysia	Ahamat *et al.* (2018)	Mohd Suki and Mohd Suki (2019)	3
	Al Mamun *et al.* (2020)		
Mexico	Leal *et al.* (2020)	Müller *et al.* (2021)	2
Poland	Caniëls *et al.* (2021)		1
South Korea	Ahn *et al.* (2020)	Park and Sohn (2018)	2
USA	Choi and Johnson (2019)	Onel (2017)	2
USA and South Korea	Van Tonder *et al.* (2020)		1
Vietnam	Giao (2020)		
Total			41

**Figure 4.**
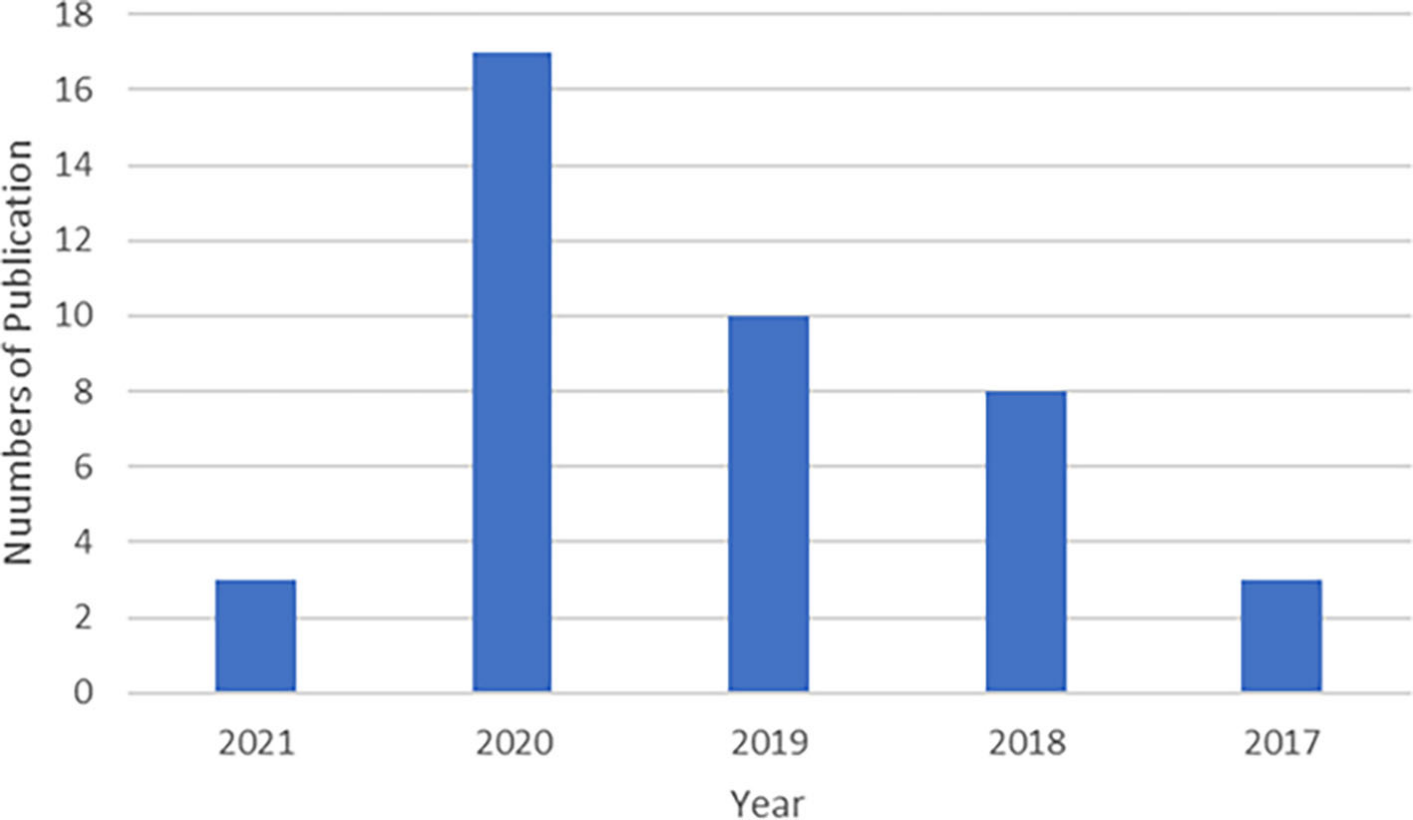
Numbers of journal produce by year.

Surveys were a typical approach to investigating the drivers (30 studies). Only one study combined qualitative and quantitative information ( Eberhart and Naderer, 2017). For qualitative studies, the research either used combination of observation and in-depth interview ( Morales-Contreras *et al.,* 2019), in-depth interview ( Giao, 2020; He *et al.,* 2021) or focus group ( Nimri, Patiar and Jin, 2020). Meanwhile, most studies (37 studies) were based on quantitative data, which was more than 11 times that of qualitative data. The research was published between 2017 and June 2021 ( Figure 4). The trend in five years’ time shows an incline of studies in GPB with 2020 obtaining the highest i.e., 12 articles. However, our final search was conducted in June 2021 with only recording four articles in mid-year, 2021 forecast a decline in GPB study as shown in Figure 4.

The articles reviewed were from various publications ( Figure 4). The majority of the articles were published in Sustainable (Switzerland) (10 studies) and Journal of Cleaner Production (eight studies). This was followed by Sustainable Production and Consumption (four studies), Business Strategy and the Environment (three studies), Journal of Retailing and Consumer Services (two studies) and one study each from another journal listed in Table 4. Table 5 shows the most cited papers in the review, where the study by Jaiswal and Kant (2018) was the most cited paper with 345 citations.

**Table 4.** Details of journal sources.

Source	Publisher	Abs Ranking	Impact Factor (IF)/Cite Score (CS)	Total number of publications
Business Strategy and the Environment	John Wiley and Sons Ltd	3	10.801(IF)	3
Ecological Economics	Elsevier	3	6.536(IF)	1
EuroMed Journal of Business	Emerald Group Publishing Ltd.	1	5.37(IF)	1
Frontiers in Psychology	Frontiers Media S.A.	1	4.232(IF)	1
International Journal of Consumer Studies	Wiley-Blackwell Publishing Ltd	2	7.096(IF)	1
International Journal of Green Economics	Inderscience Enterprises Ltd.	-	1.23 (IF)	1
International Journal of Manufacturing Technology and Management	Inderscience Enterprises Ltd.	-	0.8(CS)	1
International Journal of Productivity and Performance Management	Emerald Group Publishing Ltd.	1	3.99(IF)	1
Journal of Cleaner Production	Elsevier Ltd.	2	11.072(IF)	8
Journal of Consumer Marketing	Emerald Group Publishing Ltd.	1	2.77(IF)	1
Journal of Hospitality and Tourism Management	Elsevier BV	1	7.74(IF)	1
Journal of Islamic Marketing	Emerald Group Publishing Ltd.	-	3.70(IF)	1
Journal of Marketing Management	Taylor and Francis Ltd.	2	4.60(IF)	1
Journal of Nonprofit and Public Sector Marketing	Routledge	-	1.62(IF)	1
Journal of Retailing and Consumer Services	Elsevier Ltd.	2	10.972(IF)	2
Social Behavior and Personality	Society for Personal Research	-	1.26(IF)	1
Social Marketing Quarterly	SAGE Publications Inc.	1	2.76(IF)	1
Sustainability (Switzerland)	MDPI AG		3.889(IF)	10
Sustainable Production and Consumption	Elsevier BV	-	9.06(IF)	4

**Table 5.** The most cited publications/authors, including title, year, authors, and total citations.

No	Title	Year	Authors	Total citations
1	Green purchasing behaviour: A conceptual framework and empirical investigation of Indian consumers	2018	Jaiswal and Kant	345
2	Influences of environmental and hedonic motivations on intention to purchase green products: An extension of the theory of planned behavior	2019	Choi and Johnson	149
3	Consumers’ Sustainable Purchase Behaviour: Modelling the Impact of Psychological Factors	2019	Joshi and Rahman	84
4	Exploring pro-environmental food purchasing behaviour: An empirical analysis of Italian consumers	2018	Laureti, Tiziana; Benedetti, Ilaria	80
5	Investigating the determinants of consumers’ sustainable purchase behaviour	2017	Joshi, Yatish, Rahman, Zillur	106

Table 4 shows the details of journal sources used in this study. The highest number of publications is from Sustainability (Switzerland) (impact factor 10.801) with 10 publications, followed by Journal of Cleaner Production (impact factor 11.072) with eight publications, and Sustainable Production and Consumption (impact factor 9.06) with four publications.

Table 5 showed the most cited publications/authors in this study. Jaiswal and Kant (2018) leading the list with a total of 345 citations. The second highest total citations are article by Choi and Johnson (2019) with 149 citations, followed by Joshi and Rahman (2017) with 106 citations, Joshi and Rahman (2019) with 84 citations, and Laureti and Benedetti (2018) with 80 citations.

**Figure 5.**
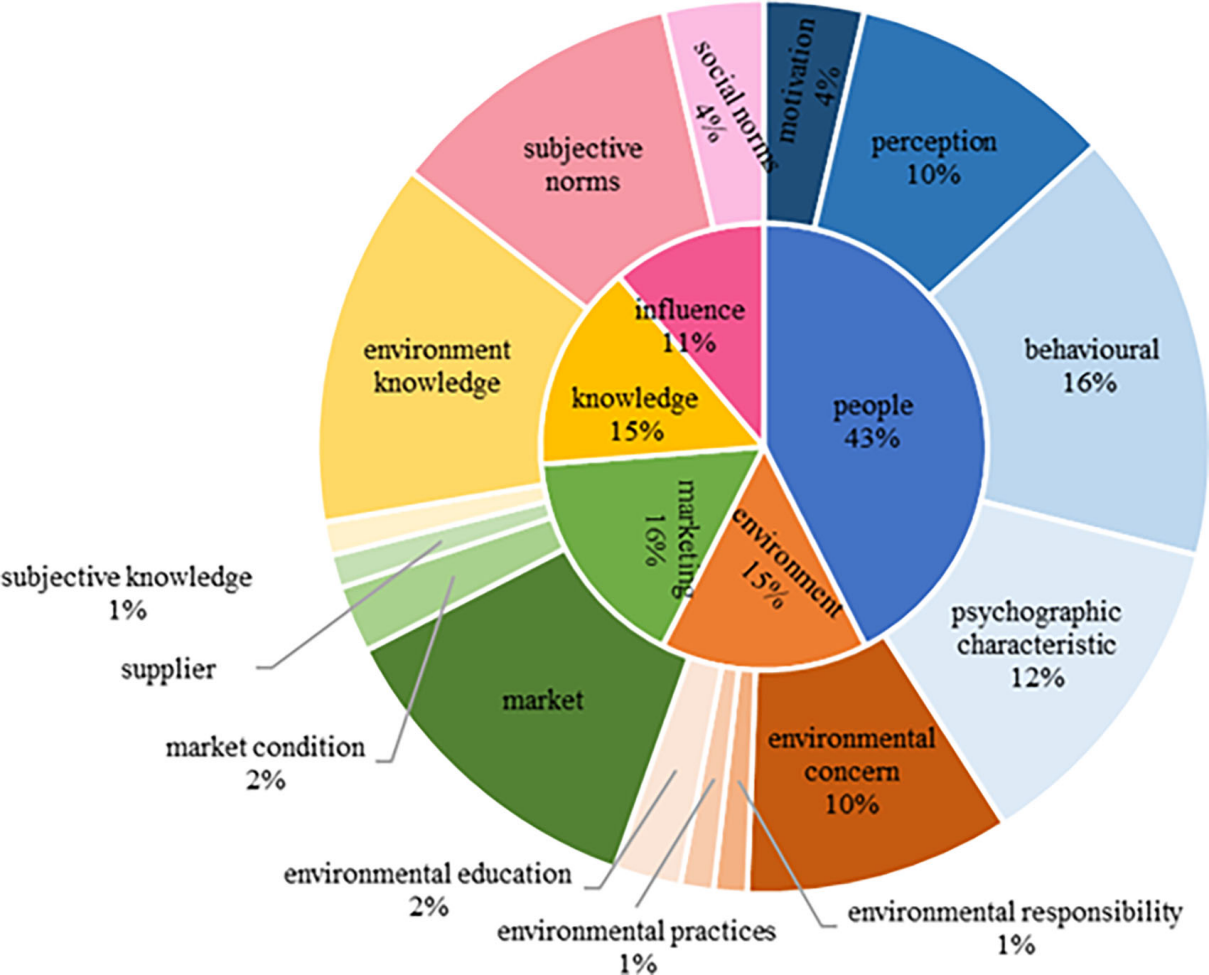
Organized systematic review papers with their respective study fields in thematic categories (Articles in total; n =41).

Five overarching themes and 15 sub-themes connected to GPB drivers were characterized by the number of incidences in the themes: People (34 papers), marketing (13 papers), knowledge (12 papers), environment (12 papers) and influence (nine papers) presented in Table 6. While Figure 5 showed the percentage of each theme’s contribution. The graph (outer circle) depicts the study’s major themes and the percentage of articles in the given axis that support the formation of thematic sub-themes.

**Table 6.** List of Themes, contribution authors and citation.

Theme	Sub-theme	Author/Year	Total citations
People	Perception	Ahmad and Zhang (2020)	8
		Al Mamun *et al.* (2020)	
		Joshi and Rahman (2019)	
		Laureti and Benedetti (2018)	
		Müller *et al.* (2021)	
		Zahan *et al.* (2020)	
		Zhang *et al.* (2019)	
		Zhang *et al.* (2018)	
	Behavioural	Ahmad and Zhang (2020)	13
		Amoako *et al.* (2020)	
		Caniëls *et al.* (2021)	
		He *et al.* (2021)	
		Nimri *et al.* (2020)	
		Onel (2017)	
		Sharma *et al.* (2020)	
		Trivedi *et al.* (2018)	
		Van Tonder *et al.* (2020)	
		Wang *et al.* (2020)	
		Yang *et al.* (2019)	
		Zahan *et al.* (2020)	
		Zhang *et al.* (2018)	
	Psychogprahic Characteristic	Al-Adamat *et al.* (2020)	10
		Ali *et al.* (2019)	
		Eberhart and Naderer (2017)	
		He *et al.* (2021)	
		Mohd Suki and Mohd Suki (2019)	
		Müller *et al.* (2021)	
		Sari *et al.* (2020)	
		Siyavooshi *et al.* (2019)	
		Uddin and Khan (2018)	
		Zhang *et al.* (2019)	
	Motivation	Choi and Johnson (2019)	3
		Eberhart and Naderer (2017)	
		Joshi and Rahman (2017)	
Environment	Environmental Concern	Al Mamun *et al.* (2020)	8
		Choi and Johnson (2019)	
		Jaiswal and Kant (2018)	
		Joshi and Rahman (2017)	
		Laureti and Benedetti (2018)	
		Troudi and Bouyoucef (2020)	
		Wang *et al.* (2020)	
		Zahan *et al.* (2020)	
	Environmental Responsibility	Joshi and Rahman (2019)	1
Marketing	Market Influence	Ahamat *et al.* (2018)	10
		Giao (2020)	
		Joshi and Rahman (2019)	
		Liao *et al.* (2020)	
		Sari *et al.* (2020)	
		Trivedi *et al.* (2018)	
		Troudi and Bouyoucef (2020)	
		Wang *et al.* (2019)	

Table 7 shows the distribution of papers that contained a stated theoretical perspective or at least a clear application of theoretical perspective. There are 22 theories mentioned in the journals. The most dominant theory is Theory of Planned Behaviour which was mentioned 15 times followed by Theory of Reason Action (seven times), Value Belief Norm (three times), and Attitude-Behaviour-Context (ABC) Theory (two times). Other theories were Theory of Normative, Social Capital Theory, grounded theory, Attitude-Intention-Behaviour, Theory of Reason Behaviour, Social Cognitive Theory, Social Adoption Theory, Theory of Consumption Value, and Stakeholder Theory.

**Table 7.** Distribution of applied theoretical perspectives.

Theory	Author	Total Citations
Theory of Planned Behaviour	Ahamat *et al.* (2018)	15
	Al Mamun *et al.* (2020)	
	Amoako *et al.* (2020)	
	Caniëls *et al.* (2021)	
	Choi and Johnson (2019)	
	Joshi and Rahman (2019)	
	Kumar *et al.* (2017)	
	Müller *et al.* (2021)	
	Nimri *et al.* (2020)	
	Onel (2017)	
	Sharma *et al.* (2020)	
	Siyavooshi *et al.* (2019)	
	Wang *et al.* (2020)	
	Yang *et al.* (2019)	
	Zahan *et al.* (2020)	
	Zhang *et al.* (2019)	
Theory Reason Action	Joshi and Rahman (2019)	7
	Joshi and Rahman (2017)	
	Sharma *et al.* (2020)	
	Zhang *et al.* (2019)	
	Sari *et al.* (2020)	
	Trivedi *et al.* (2018)	
	Troudi and Bouyoucef (2020)	
Value Belief Norm	Di Martino *et al.* (2019)	3
	Trivedi *et al.* (2018)	
	Varela-Candamio *et al.* (2018)	
Attitude-Behaviour-Context (ABC) Theory	Liao *et al.* (2020)	2
	Zhang *et al.* (2018)	
Attitude Development Theory	Park and Sohn (2018)	1
Attitude-Intention-Behaviour	Jaiswal and Kant (2018)	1
Costly Signaling Theory	Ali *et al.* (2019)	1
Goal-Framing Theory	Yang *et al.* (2019)	1
Grounded Theory	He *et al.* (2021)	1
Means-End Theory	Eberhart and Naderer (2017)	1
No theory	Leal *et al.* (2020)	1
Social Adaptation Theory	Van Tonder *et al.* (2020)	1
Social Capital Theory	De Bernardi *et al.* (2019)	1
Social Cognitive Theory	Uddin and Khan (2018)	1
S-O-R Theory	Kumar *et al.* (2019)	1
Stakeholder Theory	Witek and Kuźniar, 2021	1
Acquisition Transaction Utility Theory (ATUT)	Mohd Suki and Mohd Suki (2019)	1
Theory of Consumption Value	Wang *et al.* (2019)	1
Theory of Normative Conduct	Ahn *et al.* (2020)	1
Theory of Reasoned Behaviour	Sheng *et al.* (2019)	1
Three Moral Theories (Utilitarianism, Deontology, Virtue Ethics)	Al-Adamat *et al.* (2020)	1
WOM Theory	Ahmad and Zhang (2020)	1

Theory of Planned Behaviour and Theory of Reasoned Action are the most used theories in this findings because it is a combination of two psychological theories of health behaviour change to explain and predict human behaviour developed by Fishbein and Azjen back in 1985. ( Staats, 2004). The Theory of Reason Action (TRA) holds that one’s beliefs about behavioural outcomes and one’s evaluation of those outcomes determine attitudes toward the behaviour. The TRA then bridges the gap between attitudes and behavioural outcomes by inserting the construct of ‘intentions’, the TRA holds that intentions directly lead to behaviour ( Shahid, 2015). Theory of reasoned action (TRA) ( Ajzen and Fishbein, 1980) and the theory of planned behaviour (TPB) ( Ajzen, 1985) are the major theoretic frameworks employed by many studies to examine the various forms of ecological behaviours in western countries, including purchasing of sustainable products (Liobikienė *et al.,* 2016; Vermeir and Verbeke, 2008). While Value-Belief-Norm Theory (VBN) developed by Stern ( Stern, 2000) is one of the most quoted models for clarifying pro-environmental behaviour ( Shahid, 2015). The VBN theory links a person’s ecological worldview, assessed by the new environmental paradigm e.g., Dunlap *et al.* (2000), and environmental values (e.g., Stern and Dietz, 1994).

### Thematic analysis

In this part, five themes will be covered i.e., people, influence, environment, marketing, and knowledge topics, as well as their sub-themes, will be discussed in depth in this part.

*People*

The most dominant drivers mentioned in the selected literature is on people (34 studies). Four sub-themes were identified under this theme: motivation (three), perception (eight), behavioural (13), and psychographic characteristics (10). Choi and Johnson (2019) investigated consumer motivations using a multi-level hierarchical model that included environmental and hedonic incentives. Their findings supported the notion that environmental and hedonic incentives influence green products buying intentions. This idea is in line with Eberhart and Naderer (2017) study, which said that consumers must be in a motivating state where they feel compelled to act to achieve more sustainable consumption. Spirituality is also a motivator, and it has been discovered that instilling spiritual worth in consumers has a favourable impact on their long-term purchasing behaviour ( Joshi and Rahman, 2019).

An empirical study by Joshi and Rahman (2019) asserts that customers’ long-term purchase decisions are influenced significantly by their perceptions of their own efficacy. A significant factor in green buy intent and GPB is customers’ perceptions of their own behavioural control, according to the studies ( Müller *et al.,* 2021; Zahan *et al.,* 2020). Green products are valued by customers based on their perceived worth, and this value influences their purchasing decisions, tying the consumer’s purchasing attitudes and psychological behaviour during decision-making together (Ahmad *et al.,* 2020; Zhang *et al.,* 2019). In contrast, the negative perception towards green products such as green washing reduce customers’ purchasing intentions which lead to decrease of GPB ( Zhang *et al.,* 2018). Finding by Laureti and Benedetti (2018) suggested that purchasing behaviour was influenced by perceive behavioural control. Perceived behavioural control, on the other hand, had no effect on the study sample’s intention to purchase green skincare products ( Al Mamun *et al.,* 2020).

Green buying intention is found to be influenced by behaviour motivation indirectly. The creation process of attitude runs from behaviour motivation through behavioural intention. The internal reason of a person’s behavioural purpose is behaviour motivation ( He *et al.,* 2021). People’s motivation to buy green items is directly related to their motivation to do so. The intention to acquire green items is the internal driving force of real green product purchase behaviour. People’s real behaviour is directly determined by their intentions ( He *et al.,* 2021). A large and positive influence on green purchase intentions is exerted by a person’s perception of behavioural control ( Nimri *et al.,* 2020; Wang *et al.,* 2020; Yang *et al.,* 2019; Zahan *et al.,* 2020), subjective norms intentions ( Wang *et al.,* 2020; Yang *et al.,* 2019), attitude ( Trivedi *et al.,* 2018; Zahan *et al.,* 2020), green trust (Ahmad *et al.,* 2020) and self-identity ( Sharma *et al.,* 2020). A cross-sectional study by Sari *et al.* (2020) and quantitative study by Amoako, Dzogbenuku, and Abubakari, (2020) showed that there is a strong and positive correlation between green attitudes and purchasing behaviour. Green attitudes, on the other hand, were found to mediate GPB ( Yang *et al.,* 2019).

From the perspective of worldview, Caniëls *et al.* (2021) stated that consumer biospheric values have a beneficial influence on their goals, behaviour and experience with sustainable products reveals new information on the factors that influence sustainable consumption. The positive relationship between intention and actual behaviour confirmed from study by ( Onel, 2017). Residents’ characteristics, on the other hand, impact the degree and the relation between behaviour intention and actual behaviour as a moderating variable ( He *et al.,* 2021).

Consumer GPB was strongly influenced by psychographic traits such as values, beliefs, and attitudes ( Solomon, 2018). In general, most sustained behaviour may be explained by selfish reasons linked to a close psychological distance; when things or events are close to a person’s mind, they take on a tangible meaning, which leads to increased purchasing behaviour ( Zhang *et al.,* 2019). Besides that, people who have a high sustainability personality ( Mohd Suki and Mohd Suki 2019) and self-interest ( Eberhart and Naderer, 2017) tend to purchase green products. Granzin and Olsen (1991) found that in the context of other environmental attributes, such as green buying habits, altruism has been studied further by other researchers as a powerful predictor of environmental conservation ( Uddin and Khan, 2018). Customers in underdeveloped countries may be motivated to buy green items by self-expressive benefits ( Jahanshahi and Jia, 2018). A person’s ethical beliefs can influence their positive attitudes towards green products. As a result, moral intelligence is a critical aspect in motivating behaviour and ensuring that it is sustained ( Al-Adamat, Al-Gasawneh, and Al-Adamat, 2020) and moral obligation ( Müller *et al.,* 2021) while ( Siyavooshi, Foroozanfar, and Sharifi, 2019) empirically shows that religion value effect of sustainable purchasing behaviour.

*Environment*

A sum of 12 studies acknowledge environment as driver to GPB. Three sub-themes are environmental concern (eight studies), environmental responsibility (one study), environmental practices (one study), and environmental education (two studies).

Perceived behavioural control and attitudes are influenced by environmental concern ( Wang *et al.,* 2020). A significant influence on people’s norms and, as a result, on their green consumption has been found as environmental concern. This were supported by few studies such as Wang (2020) on hotel selection in China, Joshi and Rahman (2017) on young consumer in India, Jaiswal and Kant (2018) on India consumers, Rajadurai *et al.* (2018) on gen Y in Malaysia, Troudi and Bouyoucef (2020) on green food in Algerian, Laureti and Benedetti (2018) on green food in Italy, and Al Mamun *et al.* (2020) on green skincare products. Environmental concern, on the other hand, did not appear to have a substantial additional direct effect on purchase intention, according to studies by Choi and Johnson (2019) and Zahan *et al.* (2020). The explanation for this is that environmental concern lessened in the presence of attitude and perceived environmental effectiveness ( Choi and Johnson, 2019). Study participants’ subjective norms were found to be negatively influenced by environmental concern by Zahan *et al.* (2020). Instead, the urge to acquire green products is influenced by environmental concern in an indirect way.

According to a study by Joshi and Rahman (2019), long-term purchase decisions are influenced by customers’ desire to do their part to protect the environment. By focusing on crucial psychological aspects identified in the current study, consumers’ mindsets can be shaped, and they can be directed toward long-term purchasing. A set of complementary environmental behaviours is an-other important component in the establishment of a green purchasing policy ( Leal *et al.,* 2020), whereas environmental education is the primary driver in explaining green behaviour in an institutional context ( He *et al.,* 2021; Varela-Candamio, Novo-Corti, and Varela-Candamio *et al.*, 2018).

*Marketing*

Advertising, packaging design, and product description are all part of the marketing theme, as are customer perceptions of these tools. This theme has the second most sub-themes of any theme. i.e., market instrument (10 studies), market condition (two studies), and supplier (one study). Adverts, product quality, and technical background are the focus of the first sub-theme. Research by Sari *et al.* (2020) reveals how marketing tools, such as consumer education, affect GPB. The impact of green advertising is a crucial factor of consumer buying behaviour, according to scientific findings ( Ahamat, *et al.,* 2018). People are more inclined to acquire environmentally friendly items if they find the promotional activities interesting and beneficial ( Giao, 2020). An interesting finding by Yang *et al.* (2020) showed that media persuasion can effectively affect residents’ GPB. On the other hand, environmental advertising Liao *et al.* (2020) and context of technology He *et al.* (2021) incorporating a more balanced approach to the relationship between consumer value and environmentally friendly shopping habits.

Customer decision-making is heavily influenced by quality qualities, according to Troudi and Bouyoucef (2020). Consumers want to save the environment by paying higher fees, but they also want to satisfy their practical demands when they buy environmentally friendly products ( Wang *et al.,* 2019). Consumers may choose to buy green products based on the availability of these commodities (Ahmad *et al.,* 2018). A recent study found that consumers’ purchasing decisions are significantly influenced by information on environmentally friendly products ( Giao, 2020). Environmental crisis information efficiently evoked citizens’ environmental concern while also increasing their environmental knowledge, according to Wang *et al.* (2019) and Trivedi *et al.* (2018), allowing them to quickly form an accurate cognition about the consumption value of green products.

Study by Morales-Contreras *et al.* (2019) suggested that the main drivers to effective GPB under theme marketing are market conditions, and conflicts in customer/supplier interests. Market conditions are considered in terms of demand, prices, and margins while customer/supplier interests involve conflict with short and long-term.

*Knowledge*

A total of 12 studies with two sub-themes, i.e., subjective and environment knowledge discovered related to the theme knowledge. Most of the studies consider knowledge that positively affects the GPB. Studies by De Bernardi, Bertello, and Venuti (2019), Amoako *et al.* (2020), Kumar *et al.* (2019), Choi and Johnson (2019), Di Martino, Nanere, and DSouza, (2019), Siyavooshi *et al.* (2019), Nimri *et al.* (2020), and Troudi and Bouyoucef, (2020) believe there is a substantial correlation between environmentally conscious shopping and GPB. Park and Sohn (2018), on the other hand, emphasised that GPB was influenced directly by consumers’ subjective knowledge.

Furthermore, environmental knowledge serves as a mediator in the investigation of the link between green purchasing intentions and Doctrine of the Mean ( Sheng *et al.,* 2019). Environmental knowledge is the major antecedent that influences environmental attitude, which indirectly affects GPB, according to studies by Wang *et al.* (2020) and Uddin and Khan (2018). When it comes to green purchasing, environmental awareness has little influence, according to Jaiswal and Kant (2018) and Shatnawi, Al-Faouri, and Al-Hayari (2019). On the other side, online information sharing has been found to have a favourable impact on long-term purchasing and consumption behaviour ( De Bernardi *et al.,* 2019).

*Influence*

A total of nine research papers revealed drivers associated with the theme influence. They were divided into two sub-themes subjective norms (nine studies) and social norms (three studies). Some of papers under this theme contains both subthemes. We are more likely to engage in ecologically friendly purchasing practises if we perceive that individuals we hold in high regard (family members, close friends, etc.) support our decision to do so. As a result, higher subjective norms are likely to lead to relevant behaviour through improved behavioural intentions ( Onel, 2017). As a result, consumers’ long-term purchasing behaviour might be shaped by reinforcing subjective norms ( Joshi and Rahman, 2017). Studies by Laureti and Benedetti (2018) on Italians’ purchasing behaviour toward organic food products has echoed the same notion. However, according to Kumar *et al.* (2017), the subjective norm has no substantial relationship with purchase behaviour. Behaviour is more represented by social norms in the setting of Indian consumers’ collectivistic culture ( Bontempo and Rivero, 1992), and so a favourable norm toward environmental concerns may contribute to the disposition of related behaviour.

As moderating factors, social norms alter the degree and direction of the link between behaviour intention and actual behaviour ( He *et al.,* 2021). The “intention–behaviour” mismatch can be explained in part by factors including social environment, social conformity, and expression language ( He *et al.,* 2021). This conclusion was confirmed in a study of Korean university students by Ahn *et al.* (2020). When it comes to GPB in an emerging market, Germany’s research of organic food consumers revealed that social pressure was a key factor in determining how likely customers were to adopt green shopping habits ( Mohd Suki and Mohd Suki 2019).

## Discussion

Five themes and 15 sub-themes emerged from a thorough search of two databases for articles related to GPB drivers. Drivers on GPB form in the shape of people, environment, marketing, knowledge, and influence. The most often stated drivers in each theme were reviewed by the authors to emphasise the most common push factors for each type of driver.

We’ll start with the most frequently cited drivers from the theme people, which are the most frequently cited drivers in the review. The consumer’s behavioural characteristic was mentioned in 14 of the 41 articles. The diversity of the drivers revealed the complexities of human variables. According to our perspective, the most prevalent drivers suggest a widely occurring point, and therefore is the primary driver for long-term purchase behaviour. This corresponds to the findings of Liobikiene *et al.* (2016), who discovered that internal variables such as environmental attitude and perception of environmentally friendly behaviour are more important drivers than others. As proven in this study, the theme people comprise marketing, knowledge, environment, and influence is the most dominant theme.

As stated earlier in the descriptive analysis, most of the journals evaluated incorporate features of the Theory of Planned Behaviour (TPB), therefore it is not surprising that this theory dominates this study. According to the TPB, to anticipate an individual’s behaviour (e.g., I buy green items), one must first measure the person’s behavioural intention (e.g., I intend to purchase green products). The TPB has been used to predict and explain environmentally beneficial behaviour. TPB behavioural intentions are determined by three factors: (1) attitudes toward the behaviour, (2) subjective norms, and (3) perceived behaviour control. Furthermore, consumer motivation and characteristics may be regarded as an important factor that impacts an individual’s purchasing behaviour. In understanding green product purchasing behaviours, as part of their investigation into hedonic motives ( Choi and Johnson, 2019), they looked at how the need for novelty and adventure affects people. While spirituality acts as a motivation and influences an individual’s behaviour, it is not the same for everyone ( Joshi and Rahman, 2019).

As mentioned in the preceding paragraph, the most frequently referenced drivers in the evaluation are not from the topic influence, but from the theme people; nevertheless, this does not indicate that drivers linked to influence should be overlooked. Subjective norms are a component of external beliefs that may be used to explain public behaviour ( Groening, Sarkis and Zhu, 2018). If a person believes that others who matter to him or her approve or disapprove of his or her behaviour, he or she is more or less likely to engage in it. People use social norms as guidelines for what constitutes appropriate behaviour, assisting them in determining whether a particular behaviour is easy to employ or beneficial, and in determining what is decently correct and incorrect, as well as whether a particular behaviour is simple to employ or beneficial ( Bamberg and Möser, 2007). As a result, consumer decisions are influenced by personal norms, which are mostly influenced by the social standards of or-ganisations in which a consumer values participation ( Gleim *et al.,* 2013).

Environmental concern has also emerged as a significant component in the research of purchase intentions ( Laureti and Benedetti, 2018), and psychologically, it affects consumer attitudes, their perception of their own power over behaviour, and their own personal standards ( Asih *et al.,* 2020). Attitudes, subjective standards, and perceptions of behavioural control are all impacted by environmental concerns ( Wang *et al.,* 2020). Teksoz *et al.* (2016) discovered a substantial relationship between environmental concern and people’s views about the environment and outdoor activities. Another important psychological aspect related to the environment is environmental responsibility ( Joshi and Rahman, 2019). Individuals may become more aware of their obligation to safeguard their surroundings if they comprehend the impact of environmental degradation on humans and other living creatures and plants ( Lee, 2008). Individual responsibility may be evident in green product purchases, which indicate customers’ willingness to pay a higher price ( Verma and Chandra, 2018). Environmental education proves itself to be an effective instrument for instilling green behaviour in individuals. A wide range of elements go into environmental education, such as increasing students’ environmental consciousness, knowledge, and attitudes, as well as their capacity to identify and contribute to the solution of environmental problems ( Varela-Candamio *et al.,* 2018).

As for marketing theme, market instrument was the dominating sub-theme (10 studies). Many organisations have gone green by using green marketing strategies that target eco-conscious customers who are eager to enjoy both personal and environmental benefits. Competitive advantages can be manipulated in driving the GPB by utilising the positive environmental characteristics or the price of the green products. Green advertising is one of the most effective marketing tools for promoting environmentally friendly products and services. Green marketers that use sustainable marketing and communication methods and practises will be able to boost their environmental reputation ( Giao, 2020). Promoting environmentally friendly items is more likely to have an impact on consumers’ purchase decisions if they are more attractive and useful. Because of this, marketing campaigns must use the right channels and convenient venues for customers to learn about green products, such as supermarkets and trade events. On top of that, the quality of the products itself was the one of the marketing instruments that plays important factor in predicting GPB ( Troudi and Bouyoucef 2020; Wang *et al.,* 2019).

Through 12 studies, it has been proven that knowledge, as one of the themes, is positively associated with GPB. Several studies have found that a person’s views and behaviours are shaped in part by the information they have about a topic. Contrarily, the results of similar research on the influence of environmental knowledge on supporting behaviour under environmental settings are consistent ( De Bernardi *et al.,* 2019; Jaiswal and Kant, 2018; Nimri *et al.,* 2020). Having a positive attitude and feeling of well-being about the environment influences consumers’ shopping decisions, which in turn affects the environment ( Kumar *et al.,* 2019; Nimri *et al.,* 2020; Troudi and Bouyoucef, 2020). It has two effects on environmental intention. For starters, it may alter an individual’s environmental attitude, leading to the formation of intentions. People’s attitudes toward environmental care and awareness can be influenced by increased information ( Di Martino *et al.,* 2019; Uddin and Khan, 2018). Some researchers have found that people’s attitudes, social norms, and perceptions of behavioural control are influenced by knowledge ( Amoako *et al.,* 2020; Kim *et al.,* 2019; Liao, 2019). Green knowledge lowers customer uncertainty because credible environmental information impacts consumers’ views toward green brands ( Amoako *et al.,* 2020; Liao, 2019).

Subjective knowledge was more influential than objective knowledge when it came to green purchasing, according to Park and Sohn (2018). To promote environmentally friendly attitudes and behaviours, Park *et al.* (2018) found that objective information had an impact on subjective information, meaning that objective information should be upgraded to improve subjective information.

### Managerial implication

This review has produced a list and description of current GPB that can be used as a knowledge base for future researchers as well as guidelines for policymakers and practitioners. The findings of our study could provide useful insights into managerial decision making, i.e., informing the policy maker about which drivers are likely to influence SPB so that they provide incentives to work. For example, to boost the environment concern and responsibility via SPB, the government could educate people through media social campaigns and community services events. Policymakers and public administrators should set out a variety of effective approaches to gauging public opinion of green purchasing, as well as complementing environmental practises and legislation in place, as well as adequate resources of all sorts to encourage SPB i.e., strengthening the regulations on no plastic bag policy. Environmental conservation should be emphasised in the advertising campaigns of industrial actors, since this instils in customers the idea that they would fulfil their environmental responsibilities by choosing green lodging. SPB should be a societal attitude, not just a ‘marketable’ or ‘advertisable’ notion. In other words, citizens must freely engage in environmental protection actions and have an innate feeling of environmental responsibility while creating and consuming. Long-term improvements would occur only if sustainable consumption became a natural cognitive process and voluntary consumption behaviour. The current study’s emphasis on important psychological elements can change customers’ mindsets and guide them toward green purchasing.

Our study also provides light on current market trends and consumer behaviour in the green industry as market instrument, market condition and suppliers are the key drivers. In this sense, green product marketing strategies must be properly and strategically designed, positioned, and used in order to encourage favourable sales of green products in the market which involves the whole supply chain. Incentives for manufacturers and suppliers to embark and invest in green market should be supported by the government i.e., via grant, loan, and tax exemption. In the end, green advertising is an essential commercial communication tool for increasing customer purchase intentions, which finally leads to environmentally responsible behaviour. Finally. With every method of advertising, customer approval, as well as their subsequent environmental behaviour, are critical aspects in determining its effectiveness.

### Limitations

This study has certain flaws that have been highlighted. For starters, the results of this study derived from the method of SLR mentioned above. There are many other ways to do the SLR. In this study, we suggest studying the subject from a different SLR perspective. A different SLR method could lead to a better understanding of the drivers of GPB. This analysis excludes unpublished contributions to books, conference papers, and journals. Secondly, the analysis was confined to articles from two databases that were published during the last five years. In presenting the study of journal publications, we purposefully included references that are widely regarded as seminal. This helps to solve some of the limitations of the SLR approach and provides a more comprehensive overview of the developing area of sustainable buying behaviour. All types of articles should be considered for future research. Thirdly, consideration of longer publication period of reviewed journal may show more prominent trend in GPB. Other than that, our eligibility criteria excluded non-English papers—it is plausible that research works written in different languages would have given a salient contribution to this review. Finally, to the best of our knowledge, we considered all the eligible papers given our selection criteria, but it is possible that some papers were missed.

## Conclusion

Results of an extensive literature evaluation on GPB were presented in this study. This review contributes to the literature on GPB by providing a mapping of empirical findings associated with drivers to this action. 41 articles were analysed derived from PRISMA 2020 and MMAT 2018. Five themes and 15 sub-themes were identified. The paper’s contribution is the advancement of knowledge on GPB by highlighting the main drivers, recognising the limits, and pointing the way forward for further research. By reviewing significant publications, theories, techniques, and variables of interest in existing literature, we were able to organise and identify improvements in analytical areas of GPB research. In addition, we created a thematic theme for examining the drivers of GPB.

Based on the existing state of literature, the study proposes several potential directions for further research on the issue. First, our SLR synthesis found that the most dominant theme is about people. Consumer’s motivation, perception, behavioural and characteristic were the most influenced factors on GPB. This theme can be expanded, for example adding personal value as a sub-theme. Personal value orientations have been central to social sciences in explaining people’s motivations and behaviours ( Caniëls *et al.*, 2021). It influences environmental behaviours and the link between them. These factors are found to have different degrees of influences in explaining people’s environmental behaviours ( Steg and Vlek, 2009).

Our SLR suggests that other important drivers such as environmental concern, environmental responsibility, and environmental practices under the environment theme. Environmental concerns are frequently mentioned under the theme environment, and this is true for both developed and developing countries. In future, it is recommended to do cross-contextual research. As people are greatly influenced by their living settings and surrounding environments, these external factors are equally important as individual’s sociodemographic and socio-psychological factors when we aim to explain people’s complex environmental behaviours ( Robertson, 2018).

We discovered that a substantial number of articles on GPB use theory of planned behaviour as their primary approach. A variety of additional theories are also being utilised to support sustainable buying behaviour, with some combining two or more complementary ideas, such as Value Belief Norm and Attitude-Behaviour-Context. Lastly, the review indicates that there is an increasing trend among developing countries in GPB studies. It shows that green behaviour had gained interest among researchers in developing countries as it was already mature in developed countries. One direction of research on green purchasing intention that has received increasing academic attention concerns the power of consumers’ cultural values ( Sheng *et al.,* 2019). We suggest considering the theory of psychographics, as proposed by Wells (1975) in future study. This theory contends that values shape an individual’s lifestyle and, as a result, explain their various purchase behaviour patterns. Cultural values do, in fact, influence customer behaviour when it comes to ecologically friendly items, according to studies ( Cordano *et al.,* 2011; Riley *et al.,* 2012). Some researchers have also revealed that consumers’ lifestyles are heavily based in their cultural values ( Lee *et al.,* 2015; Nordlund and Gravill, 2002; Stern *et al.,* 1993).

## Data Availability

The data for this article consists of bibliographic references, which are included in the References section. Repository: PRISMA checklist for ‘Drivers of Green Purchasing Behaviour: A Systematic Review and a Research Agenda Drivers of Green Purchasing Behaviour: A Systematic Review and a Research Agenda. https://doi.org/10.6084/m9.figshare.24131568.v1 ( Yusoff, 2023). Data are available under the terms of the Creative Commons Zero “No rights reserved” data waiver (CC0 1.0 Public domain dedication).
