# Supplementary material for: Drivers of green purchasing behaviour: a systematic review and a research agenda
Source: F1000Res. 2023 Oct 9;12:1286. [Version 1] doi: 10.12688/f1000research.140765.1 (PMC10774633; doi:10.12688/f1000research.140765.1)
Supplement: Peer review history

# Reviewer report: Reviewer response for version 1

**DOI:** 10.5256/f1000research.154154.r219296

**Published:** 2024-01-08

**Authors:** Muhammad Syukri Abdullah

**Affiliations:** 1 Universiti Teknologi Mara, Shah Alam, Malaysia

This research is very good. However, there is a little room for improvement that can be made by the authors in order to meet with the journal requirement.

1. The authors should highlight the urgency to conduct the study in the Introduction section.
2. The authors should thoroughly discuss each variable in the LR section. The authors are highly advised to put sub-sections in the LR section to discuss and explain further. 
3. In the analysis part, the authors are highly advised to discuss on theories related to the study too. Grounded theory is very important in every research. Perhaps, one section under the Analysis part can be highlighted.

Are the rationale for, and objectives of, the Systematic Review clearly stated?

Yes

Is the statistical analysis and its interpretation appropriate?

Yes

Are sufficient details of the methods and analysis provided to allow replication by others?

Yes

Are the conclusions drawn adequately supported by the results presented in the review?

Yes

Reviewer Expertise:

Human Resource Management, Organisational Behavior, Social Finance, Marketing

I confirm that I have read this submission and believe that I have an appropriate level of expertise to confirm that it is of an acceptable scientific standard.

# Reviewer report: Reviewer response for version 1

**DOI:** 10.5256/f1000research.154154.r219297

**Published:** 2023-11-06

**Authors:** Sallehhuddin Abdullah

**Affiliations:** 1 Universiti Tunku Abdul Rahman, Cheras, Malaysia

The manuscript is recommended for indexing. The chosen focus i.e. green purchasing behaviour is timely and consistent with the development of ESG, UN 30 SDG, as well as the global commitment towards net zero carbon by 2050. The method used to conduct the study and to prepare the report is a common and widely accepted approach for systematic literature review paper. The analysis and the discussion is appropriately and well-explained.

 The only concern is the discussion on future agenda of research on green purchasing behaviour based on the given analysis. The authors may provide greater explanation and in-depth discussion about the future research agenda or new or innovative research agenda in the area of green purchasing behaviour based on this systematic literature review.

Are the rationale for, and objectives of, the Systematic Review clearly stated?

Yes

Is the statistical analysis and its interpretation appropriate?

Yes

Are sufficient details of the methods and analysis provided to allow replication by others?

Yes

Are the conclusions drawn adequately supported by the results presented in the review?

Yes

Reviewer Expertise:

Corporate social responsibility, ESG, ethics, governance

I confirm that I have read this submission and believe that I have an appropriate level of expertise to confirm that it is of an acceptable scientific standard.
